# Living with the Plagues

**DOI:** 10.3201/eid1005.031056

**Published:** 2004-05

**Authors:** Setu K. Vora

**Affiliations:** * New York Weill Cornell Medical Center, New York, New York, USA

**Figure:**
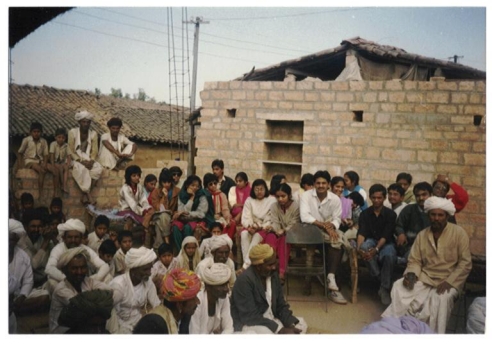
Medical students on community health visit to the remote villages of Gujarat, India.

A series of massive explosions rocked Mumbai, India, on March 12, 1993, killing at least 200 and injuring thousands. Shock, agony, anger, and fear spread from the clouds of smoke and debris. Then came corrosive anxiety—the hallmark of our age. Belief in fate and karma kept us going as we learned to live and cope with terrorism. Then, just as monsoon follows Indian summer, another tragedy came. In September, an earthquake near Latur, Maharashtra, flattened villages, killed thousands, and spawned bubonic plague. We drowned our sorrows in festivals, cricket matches, and movies, and we lived on.

I was in medical school when the next disaster struck in 1994. They came with their faces covered with handkerchiefs. Luggage in hand, they rushed from buses or trucks outside the hospital into the emergency room, where I was an intern. The first few masked patients did not attract much notice, but a roomful of them clamoring for attention and begging for tetracycline created a ruckus. Torrential rains had flooded the city of Surat on the bank of the river Tapti, and mystery pneumonia gripped the population, killing them quickly with respiratory failure. The word “plague” was all over the streets and the news. The local doctors were among the first to flee. “Diamond City” lost its luster as immigrant workers scrambled on to trains, buses, and trucks, toward their hometowns. Surat was desolate. Flights and exports from India were banned, garbage and rats ruled the streets, and panic settled in. Then, the floodwaters of the river receded, and the outbreak subsided as quickly and mysteriously as it had erupted.

The reemergence of Black Death caused much soul-searching among the intelligentsia. They blamed it on the earthquake in Maharashtra, monsoon floods, poor sanitation, and angry gods. “Foreign hand” alarmists raised the specter of bioterrorism. According to the Hindustan Times, a highly placed official, on condition of anonymity, said, “We already know that the Surat strain was not Indian. We cannot rule out the possibility of militants purchasing the organisms from a Kazakhastan company and releasing them in Surat.” Somehow, believing a disease is exotic and imported is comforting. This outbreak left 56 dead; malaria and tuberculosis, which kill thousands daily, do not generate half as much excitement or introspection—with familiarity comes tolerance. This was my first encounter with the plague.

Three years later, an internal medicine resident and first responder to a disease outbreak, I did not know if I should be proud or petrified. This responsibility was not mentioned in the residency curriculum; in fact, I do not recall seeing a curriculum. In the rain-drenched, fertile farms of southern Gujarat, sugarcane and paddy farmers were reaping more than they sowed. They were coming down with high fever, severe body pains, jaundice, and kidney failure. Leptospirosis was raging, and I was sent to care for patients at Navsari Civil Hospital. The train ride from Baroda to Navsari was my chance to learn about leptospirosis. At the hospital, I was escorted to my room adjacent to the leptospirosis ward. I was relieved to see a mosquito net over my bed. I was well-prepared to face Weil’s syndrome. After all, I had observed insertion of one peritoneal dialysis catheter and had even inserted one myself before I reached Navsari. I may have saved a few patients from uremic deaths, and many that died in spite of my efforts probably would have died of the disease anyway. I maintained a database of 71 cases, logging patients’ initial symptoms, laboratory tests, and clinical course. A few likely had other diseases, malaria or dengue fever, but we had no way of knowing right away, since sera were sent to New Delhi for testing. I never saw the results. Back in the medical college, professors were pleased with the media coverage of their generously lending resident doctors to a noble cause. My 2-week tour of duty was soon over. One month later, cerebral malaria caused by *Plasmodium* *falciparum* was the new killer stalking villages, this time in the northern districts. I was relieved to be gone by that time.

I escaped these plagues by leaving India behind for the medical world of the United States. Physicians with an accent are accepted in the U.S. healthcare system, and patients are often curious to know more about them. Patients often ask me, “Where do you come from, India or Pakistan?” This is usually followed by polite remarks, “I have always wanted to visit India,” or “I love Indian food.” I never know how to respond to the question, “What made you come to the United States?” The usual answer is, “To learn advanced medicine and research.” But I know there is more to it. I love India dearly, but I did not have the courage or the patience to deal with her unmet potential, challenges, and misplaced priorities.

I admire and envy a friend who went back to India after a brief stint at Johns Hopkins. He and other like-minded physicians now work in rural India, combating malaria, malnutrition, and maternal and infant deaths. They struggle against apathy, politics, and poverty. Like Sisyphus, they push TB and AIDS up the hill each day, only to see them roll back again each night. I chose to practice healing in a setting free of mosquitoes, politicians, charlatans, and terrorists.

When mosquito-borne West Nile virus encephalitis emerged in New York in 1999 and rapidly spread throughout the United States, it brought with it the familiar insecticide spraying and fogging, reminding me of my old home. Once again, bioterrorism was the whispered cause of this emerging disease. Then the events of September 11, 2001, scarred my adopted homeland forever. My wife saw the airplanes plunge into the towers from her safe vantage point on the West Side. The plague of terrorism had arrived, soon followed by an intentional outbreak of anthrax, which changed forever the way we handle mail and look at white powder.

Not all infectious threats we face are of exotic origin. During my fellowship in critical care, the once-familiar bugs *P. vivax*, *Entamoeba histolytica*, and even *Salmonella* almost felt benign when compared to methicillin-resistant *Staphylococcus aureus*, vancomycin-resistant *Enterococcus*, and multidrug-resistant gram-negative bacteria that lurk in shiny hospitals in the United States. Our system valiantly supports many terminally ill patients shuttling between nursing home and intensive care unit for catheter or device-related infections. These patients and their next of kin still have the right and luxury to crave the elusive cure. Within months, these patients become petri dishes, reflecting all the prevalent nosocomial pathogens in a hospital. Superbugs are also born in the United States.

The plagues may have some evolutionary role or may be designed to save us from our hubris. As predicted by Hans Zinsser, “…however secure and well-regulated civilized life may become, bacteria, protozoa, viruses, infected fleas, lice, ticks, mosquitoes, and bedbugs will always lurk in the shadows ready to pounce when neglect, poverty, famine, or war lets down the defenses” (Rats, Lice, and History; 1934). Eradicating the plagues seems futile if we do not address the factors that trigger them.

Disasters followed by plagues recur throughout the world. An earthquake in India that spawns plague, ecologic disasters in Africa that unleash Ebola, and terrorism in the United States piggybacked by anthrax make all nations vulnerable. Poverty, ignorance, hatred, and infection can breed in lands across the ocean and reach us by air to poison our existence. As a physician, I could not escape the plagues by moving away. I could only exchange them for new ones. According to the ancient Sanskrit concept of *Vasudaiva Kutumbakam*, the world is one family. We are likely to eat contaminated imported food and travel to and host visitors from hot zones of disease and terror. Heightened surveillance for imported disease may not be sustainable unless wedded to primary prevention and control of disease at its source. The plagues know no boundaries, and in our efforts to prevent them, neither should we.

